# Adenomyosis Localized in Both the Anterior and Posterior Myometrium Is Associated with Deep Rectal Endometriosis: A Retrospective Study

**DOI:** 10.3390/biomedicines12112527

**Published:** 2024-11-04

**Authors:** Konstantin Schawlochow, Nicolas Samartzis, Laurin Burla, Markus Eberhard, Dimitrios Rafail Kalaitzopoulos, Brigitte Leeners

**Affiliations:** 1 Department of Radiology, Cantonal Hospital of Schaffhausen, Geissbergstrasse 81, 8208 Schaffhausen, Switzerland; konstantin.schawlochow@spitaeler-sh.ch; 2 Department of Obstetrics and Gynecology, Cantonal Hospital of Schaffhausen, Geissbergstrasse 81, 8208 Schaffhausen, Switzerland; nicolas.samartzis@spitaeler-sh.ch (N.S.); laurinburla@hotmail.com (L.B.); markus.eberhard@bluewin.ch (M.E.); 3 Department of Reproductive Endocrinology, University Hospital Zurich, Frauenklinikstr. 10, 8091 Zurich, Switzerland; brigitte.leeners@usz.ch

**Keywords:** endometriosis, adenomyosis, magnetic resonance imaging, laparoscopy

## Abstract

Background: Endometriosis and adenomyosis are two closely related, estrogen-dependent, benign gynecological diseases. The available evidence on their common pathogenesis and association is limited and often does not address the heterogeneity of both entities. The aim of our study is to investigate the association between different types and localizations of adenomyosis and endometriosis phenotypes, using magnetic resonance imaging (MRI) and laparoscopic findings. Methods: We performed a retrospective observational study involving premenopausal women over 18 years old who underwent laparoscopic surgery for endometriosis and were pre-operatively diagnosed with adenomyosis through MRI examination at the Cantonal Hospital of Schaffhausen, Switzerland between 2011 and 2022. Results: Of 130 patients with adenomyosis, 23 (17.7%) women had adenomyosis only in the anterior wall (group 1), 38 (29.2%) only in the posterior wall (group 2), and 69 (53.1%) in both the anterior and posterior wall (group 3). Women in group 1 experienced significantly more dysuria compared to the two other groups (*p* = 0.018), while the prevalence of other pain symptoms (dysmenorrhea, dyspareunia, dyschesia) was comparable between the groups. Women in group 3 had significantly thicker anterior and posterior myometrium compared to groups 1 and 2 (*p* < 0.001). Co-existence of deep rectal endometriosis was more frequent in women from group 3 compared to groups 1 and 2 (*p* = 0.039) and in women with adenomyosis in the outer (extrinsic) compared to adenomyosis in the inner myometrium (intrinsic) (*p* < 0.001). Conclusions: This study provides evidence of an association between the localization of adenomyosis and the distribution of concomitant endometriosis. Specifically, adenomyosis localized in both the anterior and posterior wall appears to be more proliferative compared to adenomyosis found only in the anterior or posterior wall. This is indicated by its association with higher uterine volume, thicker posterior junctional zone, and greater myometrial thickness and with a higher co-existence with deep rectal endometriosis. These findings support an association between the development of specific subtypes of both entities, which represents a valuable resource for the identification of future targets for the treatment and clinical management of adenomyosis and endometriosis.

## 1. Introduction

Endometriosis and adenomyosis are two closely related, estrogen-dependent, benign gynecological diseases characterized by the proliferation of endometrial-like glands and stroma in ectopic locations [1]. Adenomyosis affects the myometrium, leading to focal or diffuse uterine enlargement, while endometriosis is characterized by the presence of endometrial tissue growth outside of the uterus [2]. Despite their different localizations, both endometriosis and adenomyosis share common pathophysiological characteristics [1] and result in detrimental disease symptoms such as pain, bleeding disorders, fatigue, and impaired fertility with a severe impact on quality of life [3,4,5,6,7].

The pathogenesis of endometriosis remains a subject of intense investigation [8,9,10,11,12,13,14]. The most widely accepted theory, the retrograde menstruation hypothesis, proposes that during menstruation, endometrial tissue flows backward through the fallopian tubes into the peritoneal cavity, where it implants and grows [15]. However, this theory alone fails to explain several critical aspects of the disease, particularly the various clinical presentations [9,16].

Adenomyosis is diagnosed in about 20% of women of reproductive age and the cardinal symptoms are heavy menstrual bleeding, particularly intense dysmenorrhea, and infertility [17,18,19]. The etiology of adenomyosis remains poorly understood, and multiple theories have been proposed to explain its development. These theories encompass both intrinsic factors, such as abnormal embryological development, genetic changes, including PIK3CA and KRAS mutations, tissue injury and repair or metaplasia, as well as extrinsic factors, including hormonal imbalances and inflammation [17,20,21]. However, the precise interplay between these factors and the molecular mechanisms underlying adenomyosis initiation and progression is still unrevealed. An increasing amount of ongoing research focuses on the common pathways of adenomyosis and endometriosis [1].

The prevalence of adenomyosis in women with endometriosis varies between 21.8% and 79% [22,23,24,25]. More in detail, Donnez et al. [26] found an association between large deep posterior endometriotic nodules and extrinsic (outer myometrium) adenomyosis, proposing that these lesions may originate from the posterior uterine or cervical wall and invade the rectovaginal space and the digestive tract. Another study based on MRI findings confirmed the association between deep endometriosis and extrinsic adenomyosis, while heavy menstrual bleeding occurred more frequently in the case of intrinsic adenomyosis [27].

As the correlation between different subtypes of adenomyosis and endometriosis is understudied and a better understanding could open new diagnostic and therapeutic approaches, the aim of our study was to investigate the association between different localizations of adenomyosis and endometriosis phenotypes using MRI imaging and intraoperative findings.

## 2. Methods

### 2.1. Study Population

We performed a retrospective observational study involving premenopausal women older than 18 years who underwent laparoscopic surgery for endometriosis diagnosis and/or therapy and were pre-operatively diagnosed with adenomyosis through an MRI examination in the context of pre-operative staging and preparing surgical approach at the Cantonal Hospital of Schaffhausen, Switzerland between 2011 and 2022. Women with suspected endometriosis that was not histologically confirmed, postmenopausal women, and women with concurrent malignancies were excluded. A thorough assessment of the clinical presentation and disease symptoms severity was performed for each patient during their medical history evaluation. Indications for laparoscopic surgery were endometriosis-associated pain and/or infertility as shown in Table 1.

### 2.2. MRI Examination

The MRI examinations were performed using an Achieva 3.0 T MRI (Philips, Amsterdam, The Netherlands). Patients were given spasmolytics (butylscopolamine) intravenously prior to the examination. The MRI protocols included native T2-weighted FSE (fast spin-echo) sequences with small FoV (field-of-view) in axial, sagittal, coronal, and oblique (also referred to as “donut sequence”) orientation, as well as T1-weighted fat-saturated (SPIR) or gradient-echo (GRE) sequence with large FoV in transverse orientation. Additionally, postcontrast T1-weighed fat-saturated (SPIR) and e-THRIVE sequences in transverse orientation were performed. In the absence of contraindications, an intravenous contrast medium was applied (standard dose of gadolinium chelate, 0.2 mL (= 0.1 mmol)/kilo bodyweight). No enema or purgatives were used.

### 2.3. Diagnostic Criteria

Based on previous meta-analysis from Rees et al. [28] we used the following criteria to diagnose adenomyosis in MRI: (1) thickening of the junctional zone (the subendometrial area of the myometrium) of at least 12 mm, (2) difference (delta) of maximum and minimum junctional zone of at least 5 mm, and (3) ratio of junctional zone to myometrium of at least 40%. At least one of the criteria had to be positive to diagnose adenomyosis [28].

**Figure 1:**
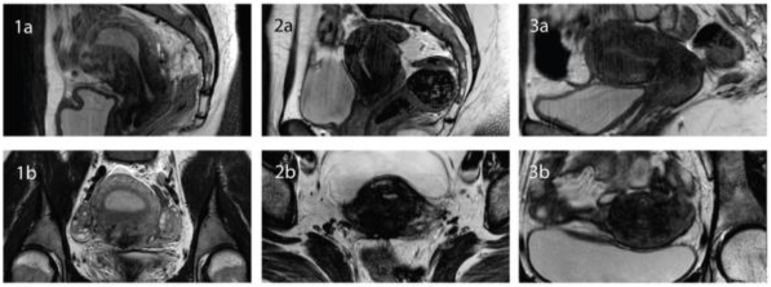
Representative T2-weighted MRI images of patients in group 1 ((**a**) sagittal and (**b**) coronal), group 2 ((**a**) sagittal and (**b**) transverse), and group 3 ((**a**) sagittal and (**b**) coronal).

According to the localization of adenomyosis, we classified all patients into three groups: adenomyosis only in the anterior myometrium (group 1), adenomyosis only in the posterior myometrium (group 2), and adenomyosis both in the anterior and posterior myometrium (group 3) (Figure 1). Additionally, we also classified the patients into intrinsic (adenomyosis in the inner myometrium) and extrinsic adenomyosis (adenomyosis of the outer myometrium) groups [29]. Uterine volume was calculated using the formula (length × width × depth × 0.52) as described by Sheth et al. [30].

Endometriosis was classified according to the revised ASRM (American Society of Reproductive Medicine) classification and the #ENZIAN classification [31,32]. The ASRM classification is the most widely used system for categorizing endometriosis, taking into account the size and subtype of endometriotic lesions as well as adhesions. Endometriosis is classified into four stages based on the total points, which indicate the severity of the condition: stage I (minimal endometriosis, 1–5 points), stage II (mild, 6–15 points), stage III (moderate, 16–40 points), and stage IV (severe, >40 points) [31]. As far as #ENZIAN classification is concerned, values for peritoneal lesions (P), and tubo-ovarian conditions (T) were assessed by laparoscopy, while ovarian endometriomas (O), deep endometriosis in the rectovaginal space (A), uterosacral ligaments and pelvic sidewall (B), deep endometriosis of the rectum (C), bladder (FB), ureter (FU), and intestine (FI) was investigated by MRI as for these compartments a high accuracy was reported [33].

### 2.4. Statistical Analysis

For statistical analysis, SPSS (version 27.0, Armonk, NY, USA: IBM Corp was used.) Percentages were calculated for categorical variables and mean and standard deviation were calculated for continuous variables. A calculation of the distribution of continuous variables was conducted with the Shapiro–Wilk test, showing normal distribution for all the variables. Chi-square was used for the comparison between categorical variables and Mann–Whitney U test was used for continuous variables. Fisher’s least significant difference test was used as a test for the correction of multiple comparisons. *p* < 0.05 was considered statistically significant.

### 2.5. Ethical Approval

The study was approved by the Cantonal Ethic Committee of Zurich (2020-02718).

## 3. Results

### 3.1. Presence and Distribution of Adenomyosis and Patient Characteristics

Out of 130 patients with adenomyosis, 23 (17.7%) women had adenomyosis only in the anterior wall (group 1), 38 (29.2%) only in the posterior wall (group 2), and 69 (53.1%) had signs of adenomyosis in both the anterior and posterior wall (group 3).

The analysis of clinical characteristics showed significant differences in parity among the three different groups (*p* = 0.011), with the highest prevalence of nulliparity in group 2 (78.9%), followed by group 1 (69.6%) and group 3 (50%) (Table 1). Women in group 2 were significantly more often nulliparous in comparison to group 3 (*p* = 0.011). The type of previous delivery did not differ significantly between the three groups (vaginal delivery and cesarean section, *p* = 0.675 and *p* = 0.193, respectively). The patients’ reported dysmenorrhea, dyspareunia, and dyschesia were also similar in women with different locations of adenomyosis. Significant difference was found with regard to the experience of dysuria (*p* = 0.018) with group 1 having the highest prevalence (26.1%), compared to group 2 (13.2%) and group 3 (4.3%). These differences remain significant after the least significant difference test (*p* = 0.048 and *p* = 0.001, respectively).

### 3.2. Characteristics of Adenomyosis

The differences in myometrial and junctional zone thickness among the three groups are presented in Table 2. In group 3, a significantly higher junctional zone thickness of the posterior uterine wall (*p* < 0.001) and thickness of both the anterior (*p* < 0.001) and posterior (*p* < 0.001) uterine walls were found in comparison to group 1 and group 2. Additionally, the uterine volume in group 3 was also higher than in the other groups (*p* < 0.001). After the least significant difference test, group 3 had significantly higher uterine volume compared to group 2 (*p* < 0.001). Regarding the localization of adenomyosis in terms of corpus vs. isthmo-cervical and intrinsic vs. extrinsic, comparable results were found among the three groups.

### 3.3. Association of the Localization of Adenomyosis and Endometriosis

Women in group 1 had significantly more frequent minimal/mild endometriosis (rASRM stage I/II) than the other groups (*p* = 0.023). The corrections for multiple comparisons also showed a significantly higher prevalence of minimal/mild endometriosis (rASRM I-II) in group 1 in comparison to group 3 (*p* = 0.006) (Table 3).

Peritoneal endometriosis, as investigated intraoperatively, was found in over 90% of all three groups (*p* = 0.072). No differences were found between the three groups regarding ovarian endometriomas (#ENZIAN O) (*p* = 0.053 and *p* = 0.715 for the left and right sides, respectively) and adnexal adhesions (#ENZIAN T) (*p* = 0.290 and *p* = 0.903 for the left and right sides, respectively).

The three groups had similar #ENZIAN A (rectovaginal space) and B (uterosacral ligaments and pelvic sidewall) and #ENZIAN FI (intestine, mainly of the sigmoid colon) prevalence. However, significantly more cases of deep endometriosis of the rectum (#ENZIAN C) were found in group 3 compared to the other groups (*p* = 0.039). The least significant difference test showed that group 3 had more rectal endometriosis in comparison to group 1 (*p* = 0.019).

Extrinsic adenomyosis was significantly associated with deep rectal endometriosis (#ENZIAN C) (*p* < 0.001). Adenomyosis located in the corpus was significantly associated with adhesions of the adnexa of the left side (*p* = 0.034) compared to isthmo-cervical adenomyosis (Table 3).

## 4. Discussion

By categorizing adenomyosis based on localization, this study identified distinct differences in clinical characteristics and the co-occurrence of endometriosis among the various subtypes.

Women with adenomyosis only in the anterior myometrium had significantly more frequently minimal or mild endometriosis (rASRM stage I/II), which is in accordance with results from Shi et al. [34]. The presence of dysuria was highest in the case of anterior adenomyosis only compared to the other groups, although the prevalence of bladder endometriosis was comparable across all groups. Dysuria in these patients could potentially be amplified through the closely localized inflammatory environment of the anterior adenomyosis, causing painful bladder syndrome. To the best of our knowledge, there is no evidence of the association between adenomyosis and painful bladder syndrome; however, epidemiological data found a significant association between endometriosis and painful bladder syndrome in comparison to women without endometriosis (RR 3.74 (95% CI = 1.76–7.94) [35]. In our population, deep endometriosis of the bladder was found in 34.8% of women with anterior adenomyosis. A previous study examining the correlation between anterior extrinsic adenomyosis and bladder endometriosis, found a co-existence in 48.7%, similar to women without anterior extrinsic adenomyosis [36]. The authors concluded that since only half of the patients with deep endometriosis of the bladder had focal extrinsic adenomyosis of the anterior uterine wall and there are also women with bladder endometriosis but no adenomyosis, a direct invasion of adenomyotic lesions is unlikely to be the sole pathophysiological mechanism for the development of bladder nodules [36]. This conclusion is also supported by our results. Some researchers have proposed that a cesarean section may also be a risk factor for the development of bladder nodules; however, in our study, we found no increased frequency of bladder endometriosis in patients with a history of cesarean sections, findings which were in concordance with Marcellin et al. [37]. Besides the theory of the iatrogenic and infiltration per continuitatem, there are further theories for the development of bladder endometriosis. Intraperitoneal endometriotic cells may penetrate after retrograde menstruation or originate from the Mullerian remnants located in the vesicouterine septum [38,39]. Fedele et al. presented one case of bladder endometriosis with the involvement of the parametrium, but without adenomyosis or peritoneal endometriosis, supporting the stem cell theory [40].

Women with adenomyosis only in the posterior myometrium were significantly more often nulliparous than the other groups, and had a higher rate of primary infertility, although this difference was not statistically significant. Women with adenomyosis in the posterior myometrium had a higher prevalence of dysmenorrhea, dyspareunia, and dyschesia, but again this difference did not reach statistical significance. The posterior localization was associated with more severe stages (III/IV) of endometriosis according to the rARSM classification than anterior adenomyosis. The above results underline that the extent of endometriosis does not always correlate with the severity of symptoms. In agreement with our findings, Shi et al. [34] found a higher rate of nulliparity in the case of posterior adenomyosis. Later follow-up showed a higher risk of obstetrical complications such as placenta previa, placenta accreta, preeclampsia, and preterm birth in their group of women with posterior adenomyosis compared to other localizations of adenomyosis [34].

Women with adenomyosis localized in both the anterior and posterior uterine wall had the significantly highest uterine volume compared to the two other groups. This subtype of adenomyosis was associated with a thicker junctional zone and a greater difference between the minimal and the maximal junctional zone in the posterior wall compared to women with adenomyosis exclusively located in the posterior wall. The highly proliferative nature of this subtype of adenomyosis is also underlined by the fact that these women have a thicker posterior myometrium and a higher prevalence of rectal endometriosis (#ENZIAN C), which possibly indicates a posterior infiltration of eutopic endometrium into the uterine wall and through the serosa to the anterior wall of the rectum. A previous study examining the clinical characteristics of women with different localizations of adenomyosis showed that women with adenomyosis of both the anterior and posterior uterine wall had statistically higher ASRM scores, more ovarian endometriomas, and more pelvic adhesions; however, uterine volume, myometrial thickness, junctional zone, and co-existence of rectal endometriosis were not examined [34].

The co-existence of endometriosis and adenomyosis of the outer posterior myometrium varies between 97% and 66.3% in previous studies [26,41]. In our population, we identified 26 women with extrinsic posterior adenomyosis, all except one of whom (96.2%) had deep endometriosis of the rectum (#ENZIAN C). Some authors have proposed that endometriotic lesions may invade through the posterior uterine or cervical wall into the rectovaginal space and digestive tract, describing this as an “inside to outside” mechanism [19], while others have suggested that menstrual debris in the pelvic cavity could lead to adhesions and obliteration of the pouch of Douglas, potentially infiltrating the posterior uterine wall from outside the uterus referred to as an “outside to inside” pathophysiological mechanism [41].

Various authors have addressed the question of whether adenomyosis and endometriosis represent one entity or not [1,42,43,44,45,46,47]. In a review, Bulun et al. demonstrated the pathophysiological similarities between endometriosis and adenomyosis [1]. Apart from their common resemblance to eutopic endometrium, oligoclonality has been found in both entities with the same driver mutations, of which KRAS is the most frequent [1]. In adenomyosis, the theory of the invagination of glands into the myometrium is supported by the occurrence of KRAS mutations in the adjacent basal layer of the endometrium [21]. On the histological level, both adenomyosis and deep endometriosis have excessive macrophage accumulation, fibrosis, and irregular angiogenesis [48]. Further pathophysiological mechanisms including alterations in hormonal receptors, epithelial–mesenchymal transition, and smooth muscle metaplasia, have been reported in both endometriosis and adenomyosis [47].

This study enrolled patients who underwent both MRI and laparoscopy, with endometriosis histologically confirmed. All MRIs were conducted by radiologists specialized in gynecologic imaging, pre-operative interviews, and clinical data collection were performed by gynecologists. However, it is crucial to acknowledge the limitations of our monocentric and retrospective study. It cannot be excluded, that the indications for endometriosis surgery might have induced selection bias. Although MRI is one of the most accurate non-invasive diagnostic methods for adenomyosis [28], the proposed diagnostic criteria have a sensitivity ranging from 65% to 93% and a specificity from 85% to 93%. To date, there is no widely accepted consensus on the diagnostic criteria for adenomyosis in MRI. Although the most widely accepted diagnostic finding for adenomyosis is a junctional zone thickness of at least 12 mm, some authors proposed that this criterion should be used with caution, as the junctional zone can also be thickened by other pathologies, and women with adenomyosis can have a normal junctional zone [49,50].

## 5. Conclusions

This study provides evidence of an association between the localization of adenomyosis and the distribution of concomitant endometriosis. Specifically, adenomyosis localized in both the anterior and posterior wall appears to be more proliferative compared to adenomyosis found only in the anterior or posterior wall. This is indicated by its association with higher uterine volume, thicker posterior junctional zone, and greater myometrial thickness. Additionally, this subtype of adenomyosis is linked to a higher prevalence of deep rectal endometriosis (#ENZIAN C). As far as the other subtypes of adenomyosis are concerned, anterior adenomyosis is associated with the existence of dysuria, while posterior adenomyosis was found more often in nulliparous women. These findings support clinical differences between the subtypes of adenomyosis and an association between the development of specific subtypes of both entities which represent a valuable resource for the identification of future targets for the treatment and clinical management of adenomyosis and endometriosis.

## Figures and Tables

**Table 1:** Clinical characteristics of the population.

	Group 1 (n = 23)	Group 2 (n = 38)	Group 3 (n = 69)	*p*-Value
Age (years) *	36.5 (SD 5.6)	33.5 (SD 6.4)	36.7 (SD 6.6)	0.092
BMI (kg/m^2^) *	22.8 (SD 5.3)	23.2 (SD 3.8)	23.8 (SD 5.9)	0.385
**Parity**				0.747
**Nullipara**	16 (69.6%)	30 (78.9%)	35 (50.7%)	**0.011**
**Primipara**	3 (13.0%)	4 (10.5%)	16 (23.2%)	0.211
**Multipara**	4 (17.4%)	4 (10.5%)	18 (26.1%)	0.148
**Vaginal birth**				0.675
**0**	18 (78.3%)	31 (81.6%)	52 (75.4%)	0.759
**1**	3 (13.0%)	5 (13.2%)	6 (8.7%)	0.720
**2 or more**	2 (8.7%)	2 (5.3%)	11 (15.9%)	0.228
**Caesarean**				0.193
**0**	20 (87.0%)	34 (89.5%)	50 (72.5%)	0.071
**1**	2 (8.7%)	4 (10.5%)	15 (21.7%)	0.181
**2**	1 (4.3%)	0 (0%)	4 (5.8%)	0.325
**Infertility**				0.262
**primary**	5 (21.7%)	13 (34.2%)	15 (21.7%)	0.331
**secondary**	0 (0%)	2 (5.3%)	9 (13.0%)	0.105
**Dysmenorrhea**	17 (73.9%)	35 (92.1%)	58 (84.1%)	0.123
**Dyspareunia**	10 (43.5%)	18 (47.4%)	27 (39.1%)	0.900
**Dysuria**	6 (26.1%)	5 (13.2%)	3 (4.3%)	**0.018**
**Dyschezia**	7 (30.4%)	19 (50%)	27 (39.1%)	0.351

Group 1: adenomyosis only in the anterior myometrium, Group 2: adenomyosis only in the posterior myometrium, and Group 3: adenomyosis both in anterior and posterior myometrium, BMI: body mass index. SD: standard deviation. * Data shown as mean (SD). Chi-square was used for the comparison between categorical variables and Mann–Whitney U test was used for continuous variables.

**Table 2:** MRI characteristics of adenomyosis.

	Group 1	Group 2	Group 3	*p*-Value
	n = 23	n = 38	n = 69	
**Uterine dimension**				
**Uterine volume (cm^3^)**	92.1 (SD 41.8)	68.3 (SD 29.5)	127.6 (SD 105.9)	**<0.001**
**Anterior wall**				
**Wall thickness (mm)**	19.6 (SD 4.7)	14.9 (SD 2.6)	20.1 (SD 5.1)	**<0.001**
**JZ max thickness (mm)**	13.5 (SD 2.9)	7.6 (SD 1.8)	13.1 (SD 3.4)	**<0.001**
**JZ delta thickness (mm)**	8.7 (SD 2.9)	3.2 (SD 1.4)	8.1 (SD 2.8)	**<0.001**
**Posterior wall**				
**Wall thickness (mm)**	18.7 (SD 3.8)	18.8 (SD 4.6)	23.2 (SD 7.7)	**<0.001**
**JZ max thickness (mm)**	7.8 (SD 1.6)	13.45 (SD 2.4)	16.0 (SD 6.4)	**<0.001**
**JZ delta thickness (mm)**	3.2 (SD 1.3)	8.4 (SD 2.0)	10.6 (SD 5.9)	**<0.001**
**Adenomyosis localization**				
**Intrinsic**	21 (91.3%)	32 (84.2%)	49 (71%)	0.072
**Extrinsic**	2 (8.7%)	6 (15.8%)	20 (29%)	0.072
**Adenomyoma**	6 (26.1%)	4 (10.5%)	17 (24.6%)	0.179
**Corpus**	14 (60.9%)	25 (65.8%)	56 (81.2%)	0.080
**Isthmocervical**	20 (87%)	34 (89.5%)	61 (88.4%)	0.956

Group 1: adenomyosis only in the anterior myometrium, Group 2: adenomyosis only in the posterior myometrium, Group 3: adenomyosis both in anterior and posterior myometrium, SD: standard deviation. JZ: junctional zone. JZ max: maximum thickness of junctional zone. Chi-square was used for the comparison between categorical variables and Mann–Whitney U test was used for continuous variables. Values are in mean (standard deviation) or number of cases (percentage of entire group). Some characteristics did occur simultaneously, thus, total cases can exceed 100%. JZ delta: difference of maximum and minimum thickness of junctional zone.

**Table 3:** Involvement of the different compartments according to the #ENZIAN classification.

			Group 1	Group 2	Group 3	*p*-Value
			n = 23	n = 38	n = 69	
**#ENZIAN**						
**P**			22 (95.7%)	35 (92.1%)	69 (100%)	0.072
**O**		**left**	20 (87.0%)	36 (94.7%)	61 (88.4%)	0.053
		**right**	19 (82.6%)	34 (89.5%)	59 (85.5%)	0.715
**T**		**left**	16 (69.6%)	29 (76.3%)	58 (84.1%)	0.290
		**right**	16 (69.6%)	27 (71.1%)	51 (73.9%)	0.903
**A**			13 (56.5%)	22 (57.9%)	52 (75.3%)	0.097
**B**		**left**	11 (47.8%)	14 (36.8%)	36 (52.2%)	0.097
		**right**	13 (56.5%)	11 (28.9%)	35 (50.7%)	0.085
**C**			10 (43.5%)	21 (55.3%)	49 (71.0%)	**0.039**
**FB**			8 (34.8%)	21 (55.3%)	41 (59.4%)	0.731
**FU**			1 (4.3%)	4 (10.5%)	14 (20.3%)	0.202
**FI**			15 (65.2%)	31 (81.6%)	55 (79.7%)	0.278
**rASRM**						
**I/II**			8 (34.8%)	7 (18.4%)	7 (10.1%)	**0.023**
**III/IV**			15 (65.2%)	31 (81.6%)	62 (89.9%)	

Group 1: adenomyosis only in the anterior myometrium, Group 2: adenomyosis only in the posterior myometrium, and Group 3: adenomyosis both in anterior and posterior myometrium. Numbers of patients (and percentage according to the population in each category) with involvement of each #ENZIAN compartment and rASRM classification (revised American Society of Reproductive Medicine) stage I (minimal), stage II (mild), stage III (moderate) and stage IV (severe). *p*-values were calculated with the Chi-square test. #ENZIAN classification P: peritoneal endometriosis, T: tubo-ovarian conditions, O: ovarian endometriomas, A: deep endometriosis in the rectovaginal space, B: uterosacral ligaments and pelvic sidewall, C: deep endometriosis of the rectum, FB: bladder, FU: ureter and FI: intestine.

## Data Availability

The data presented in this study are available on request from the corresponding author. The data are not publicly available due to privacy restrictions.
